# Supplementary figures and images for: CCNB2 and AURKA overexpression may cause atypical mitosis in Japanese cortisol-producing adrenocortical carcinoma with TP53 somatic variant
Source: PLoS One. 2020 Apr 14;15(4):e0231665. doi: 10.1371/journal.pone.0231665 (PMC7156056; doi:10.1371/journal.pone.0231665)

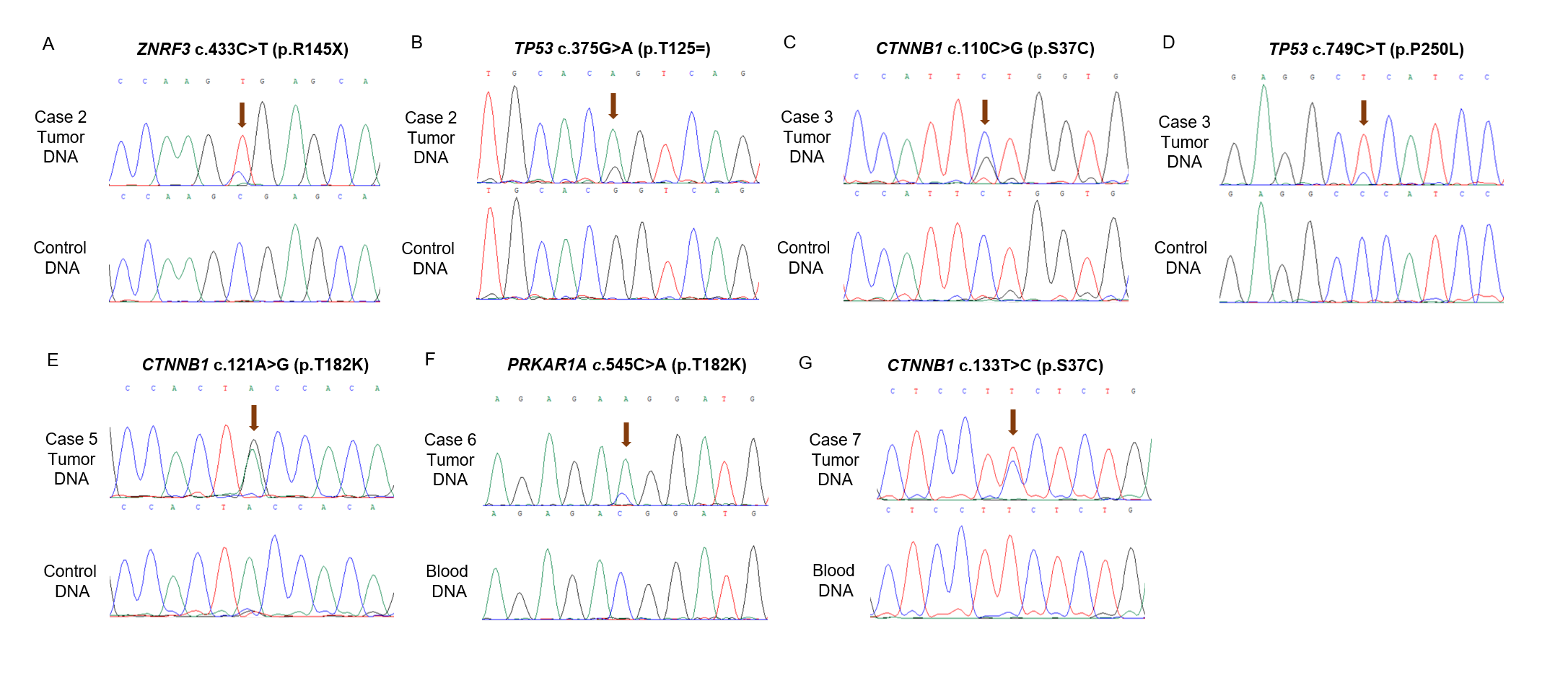

Supplement: S1 Fig — Sanger sequencing results are shown under the reference nucleotide sequences. The upper electropherograms show the sequencing results from the ACC sample, and arrows indicate the altered nucleotides. The lower electropherograms show the sequencing results for the references. Cases 6 and 7 used the patients’ own blood samples as references (indicated as blood DNA in the figure), and in other cases, healthy adult blood samples were used as the references (indicated as control DNA in the figure). (A) ZNRF3 c.433C>T (p.R145X) variant in case 2, (B) TP53 c.375G>A (p.T125 = ) variant in case 2, (C) CTNNB1 c.110C>G (p.S37C) variant in case 3, (D) TP53 c.749C>T (p.P250L) variant in case 3, (E) CTNNB1 c.121A>G (p.T41A) variant in case 5, (F) PRKAR1A c.545C>A (p.T182K) variant in case 6, and (G) CTNNB1 c.133T>C (p.S45P) variant in case 7. (TIF) [file pone.0231665.s001.tif]

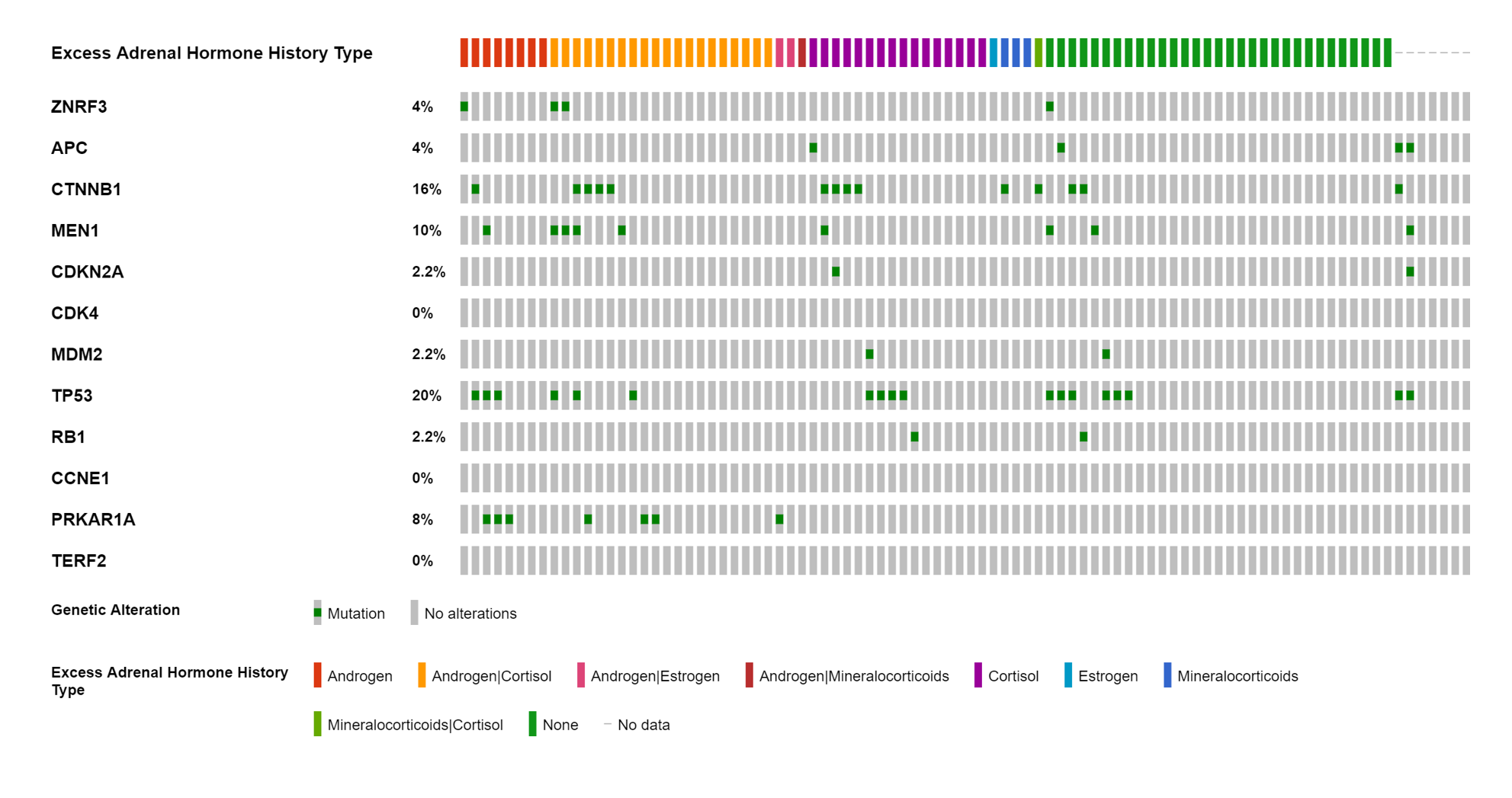

Supplement: S2 Fig — Occo Print was used to evaluate hormone excess in 83 cases of ACC from the TCGA Provisional dataset and summarize the presence or absence of variants in the 12 candidate genes of this study. "Mutation" in Occo Print is synonymous with "variant" in this article. (TIF) [file pone.0231665.s002.tif]

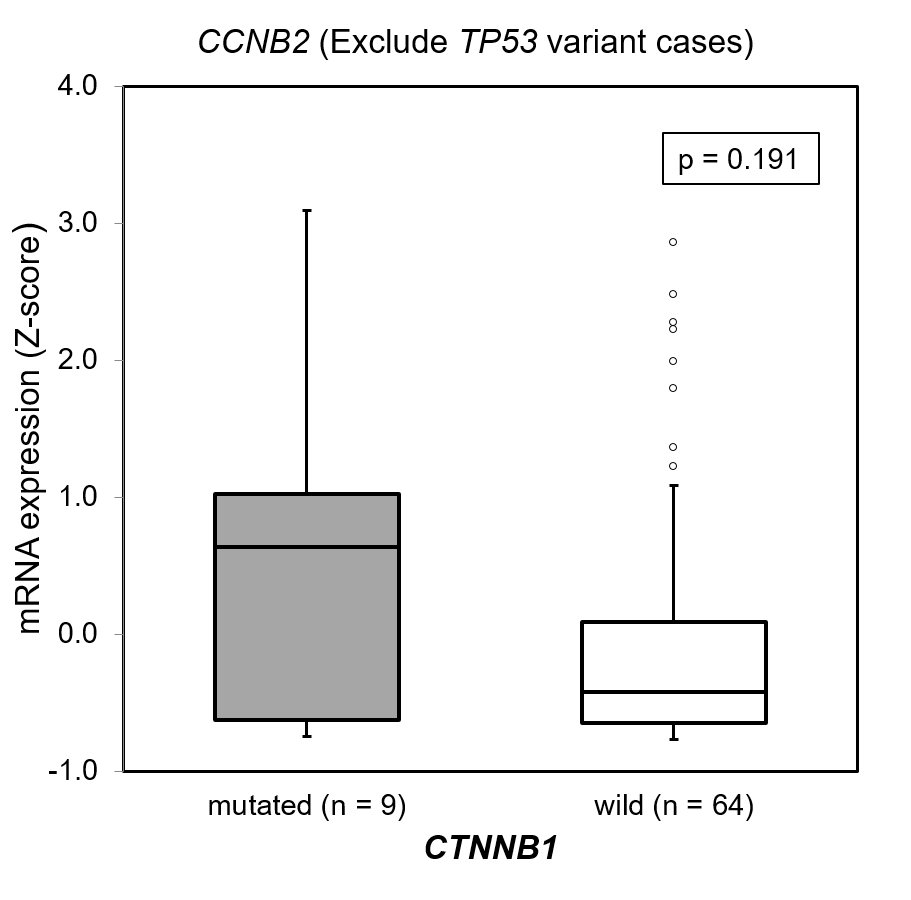

Supplement: S3 Fig — When the analysis was performed excluding 4 cases of TP53 co-mutated cases, the significant difference in CCNB2 mRNA expression between CTNNB1 mutated cases and wild type disappeared (P = 0.191). In the box plots, bounds of the box span from the first quartile (Q1) to the third quartile (Q3), and the center line represents the median. The lower whisker extends up to [Q1 − 1.5 × (Q3 − Q1)] and upper whisker extends up to [Q3 + 1.5 × (Q3 − Q1)]. (TIF) [file pone.0231665.s003.tif]

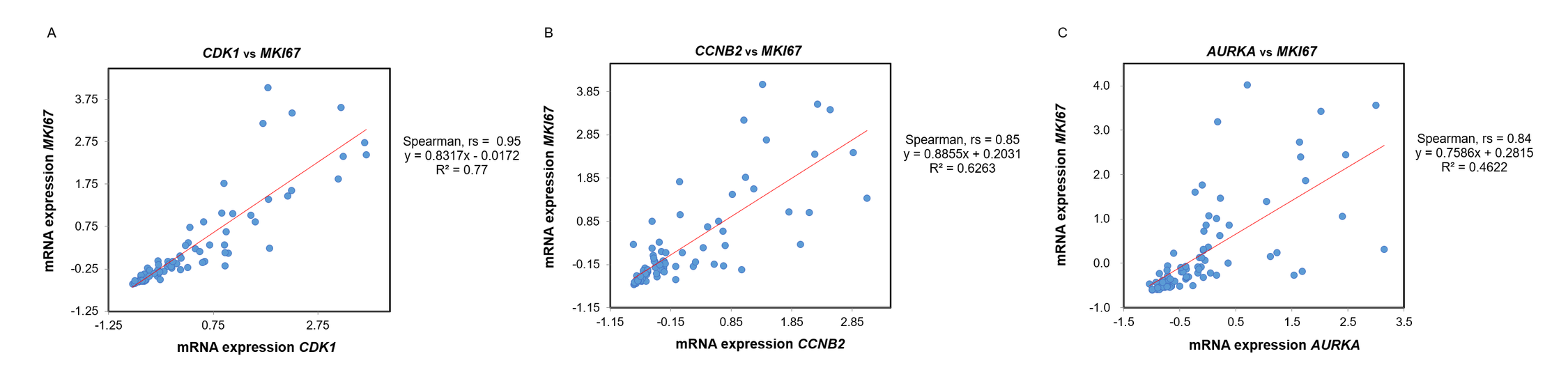

Supplement: S4 Fig — (A) CDK1 mRNA expression in ACC was positively correlated with that of MIKI67 mRNA expression (Spearman’s rank, rs = 0.95, p < 0.001). (B) CCNB2 mRNA expression in ACC was positively correlated with that of MIKI67 mRNA expression (rs = 0.85, p < 0.001). (C) AURKA mRNA expression in ACC was positively correlated with that of MIKI67 mRNA expression (rs = 0.84, p < 0.001). (TIF) [file pone.0231665.s004.tif]
