# Supplementary material for: CCNB2 and AURKA overexpression may cause atypical mitosis in Japanese cortisol-producing adrenocortical carcinoma with TP53 somatic variant
Source: PLoS One. 2020 Apr 14;15(4):e0231665. doi: 10.1371/journal.pone.0231665 (PMC7156056; doi:10.1371/journal.pone.0231665)
Supplement: Attachment — Submitted filename: Response to Reviewers.docx [file pone.0231665.s010.docx]

Dr. Sumitra Deb, PhD.

Academic Editor

PLOS ONE

U.S. Headquarters PLOS 1160 Battery Street Koshland Building East, Suite 225 San Francisco, CA 94111 United States

Re; Manuscript ID: PONE-D-20-01265

Dear Dr. Sumitra Deb, PhD.

Thank you very much for your e-mail and review of the manuscript (PONE-D-20-01265) that we sent on 14 January 2020. We appreciate a reviewer for providing constructive comments regarding the improvement of the original manuscript.

We are sending a new file of our revised manuscript. All changes have been made in response to the reviewers’ suggestion, and itemized response to the reviewer’s comments are also attached.

The revision includes a number of positive changes. Based

on your journal requirements, we:

• Added how we had obtain consent from participants in our ethics statement.

•Stopped using the phrase “data not shown” and created additional supplemental figures for this part of the data.

Response to Reviewer #1:

Comment:

Q1. Table-1, 2, 3: some columns are missing in the current version. Please upload again.

A1. I apologize for not being able to show the whole table in the original manuscript. In accordance with Reviewer #1 comment, the font size has been adjusted for Tables 1 and 3 so that all columns are visible in the revised manuscript. Table 2 has been changed to be shown as Figure 1 in the revised manuscript.

Q2. Line 104: The authors should explain the rationale for interrogating only the selected genes in this study.

A2. We sincerely accept comments made by Reviewer #1. In accordance with Reviewer #1 comment, we have added Line 106-119 in the revised manuscript explaining the reason why we selected the 12 genes analyzed as candidate genes this study.

One of our objectives of this study was to investigate the factors for poor prognosis of adrenocortical carcinoma (ACC) and to consider the potential for treatment. First, we selected ten candidate genes of TP53-RB1 and Wnt pathways, to which *TP53* and *CTNNB1* belong. *TP53* and *CTNNB1* somatic variant was reported as a poor prognostic factor in the previous report. We speculated that somatic variants of genes involved in these two genes may also contribute to poor prognosis. Because these candidates ten genes variants were detailed in a previous paper, they were also suitable for comparative studies between Japanese and other races. We further selected *PRKAR1A*, since it is associated with cortisol production, which is a factor of poor prognosis. We also selected *TERF2*, which may be associated with disease progression due to shortened telomere length, among ACC driver genes.

Q3. Line 293-299: I did not find supporting evidence in literature to attribute pathogenicity to *PRKAR1A* c.545C>A (p.T182K) variant. In silico tools in by themselves are not sufficient to attribute pathogenicity to a variant. The reasons listed by the authors to consider this variant as pathogenic are insufficient and in the absence of functional evidence, this variant would best be considered as a “variant of uncertain significance”. The reference to the variant in Clinvar is for *PRKAR1A*:c.545C>T and not c.545C>A as reported here. The authors come to similar conclusion in the discussion, and hence it is not reasonable to list it as a pathogenic variant.

A3. We sincerely accept comments made by Reviewer #1.　As suggested, since *PRKAR1A*:c.545C>T and c.545C>A are not the same, the text describing references to variants of Clinvar has been deleted. Also, we have changed Table S5 because we agree it is correct to classify the *PRKAR1A*:c.545C>A (p.T182K) variant as "variant of uncertain significance".

Regarding to the variant of the *PRKAR1A*:c.545C>A (p.T182K), it is a de novo somatic variant, a very rare variant with no registration such as gnomAD, and its pathogenic significance is predicted by in silico prediction. The pathogenicity of some missense variants of the cAMP binding domain A, such as c.547G>T (p.A183Y), c.638C>A (p.213D), and c.438A>T (p.R146S) have been demonstrated in vitro, as shown in the new reference 41 and 42. The in silico prediction of those variants, which was proven to be pathogenetic in vitro, were ”Probably damaging” or ”Benign”. The *PRKAR1A*:c.545C>A (p.T182K) variant is likely to affect cAMP binding domain A and which will affect the enzyme function of PRKAR1A through conformational changes. It is presumed that the change enhances PKA activity and affects tumor formations. The *PRKAR1A* gene is one of the ACC driver genes, and variants in this gene are presumed to be clinically significant, especially in ACC. Based on the above, we have identified the *PRKAR1A*:c.545C>A (p.T182K) variant in this study as a potential variant of carcinogenesis.

We have added the above content to LINE 559-575 and references 41 and 42 as a new reference in the revised manuscript.

Certainly, as pointed out by Reviewer #1, the effect on the actual carcinogenesis will need to be evaluated for the functional studies of the variant. We are aiming to demonstrate the actual functional evaluation of the *PRKAR1A*:c.545C>A (p.T182K) variant in the future.

Q4. Also since *ZNRF3* is an uncommon variant, the authors should consider discussing the clinical significance and role in pathogenicity in more detail.

A4. In accordance with Reviewer #1 comment, we have added a detailed discussion of the clinical significance and role in the pathogenicity of *ZNRF3*: c.433C>T (p.R145X) variant at LINE 576-588 in the revised manuscript. The *ZNRF3*:c.433C>T (p.R145X) variant was a nonsense variant of the transmembrane domain that has been reported to have a high potential for affecting pathogenicity in previous reports. It is speculated that this variant may upregulate the Wnt pathways when variant position are considered. This variant is a very rare variant that is not registered in gnomAD and its pathogenic significance was predicted by in silico prediction.

Q5. Line 331: atypical mitotic figures were positive in all of the cases with *TP53* somatic variants and Ki-67 index ≥ 10%. I would need to see the complete Table-2 for verification of this.

A5. As indicated, in the revised manuscript, we changed Table2 to Figure1 so that you can check the contents of Table2 that Reviewer #1 pointed out.

Figures and Table:

To improve the manuscript according to the Reviewers’ comments, we have changed figure composition as described below:

Previous Table 2 has been changed to Figure 1.

Previous Table 3 has been moved to Table 2.

Previous Figure 2 has been moved to Figure 3.

Previous Figure 3 has been moved to Figure 4.

Previous Figure 4 has been moved to Figure 5.

Previous Figure 5 has been moved to Figure 6.

Previous Figure 6 has been moved to Figure 7.

New Figure has been inserted as Figure S3.

Previous Figure S3 has been moved to Figure S4.

Again, thank you for giving us the opportunity to strengthen our manuscript with your valuable comments and queries. We have worked hard to incorporate your feedback and hope that these revisions persuade you to accept our submission.

Sincerely,

Miho Yamashita: M.D., Ph.D.

Department Internationalization Center, Hamamatsu University School of Medicine.1-20-1 Handayama, Higashi-ku, Hamamatsu city, Shizuoka, Japan 431-3192.Telephone number; +81-53-435-2263 facsimile number; +81-53-435-2354 E-mail; mihojy@hama-med.ac.jp
